# Bioengineering thermodynamics of biological cells

**DOI:** 10.1186/s12976-015-0024-z

**Published:** 2015-12-01

**Authors:** Umberto Lucia

**Affiliations:** Dipartimento Energia, Politecnico di Torino, Corso Duca degli Abruzzi 24, 10129 Torino, Italy

**Keywords:** Entropy generation, Exergy, Irreversibility, Living systems, Medicine and biochemistry thermodynamics, Membrane transport

## Abstract

**Background:**

Cells are open complex thermodynamic systems. They can be also regarded as complex engines that execute a series of chemical reactions. Energy transformations, thermo-electro-chemical processes and transports phenomena can occur across the cells membranes. Moreover, cells can also actively modify their behaviours in relation to changes in their environment.

**Methods:**

Different thermo-electro-biochemical behaviours occur between health and disease states. But, all the living systems waste heat, which is no more than the result of their internal irreversibility. This heat is dissipated into the environment. But, this wasted heat represent also a sort of information, which outflows from the cell toward its environment, completely accessible to any observer.

**Results:**

The analysis of irreversibility related to this wasted heat can represent a new approach to study the behaviour of the cells themselves and to control their behaviours. So, this approach allows us to consider the living systems as black boxes and analyze only the inflows and outflows and their changes in relation to the modification of the environment. Therefore, information on the systems can be obtained by analyzing the changes in the cell heat wasted in relation to external perturbations.

**Conclusions:**

The bioengineering thermodynamics bases are summarized and used to analyse possible controls of the calls behaviours based on the control of the ions fluxes across the cells membranes.

## Background

Nature, from a physical, biological, chemical and mathematical point of view, is a complex system, while from an engineering point of view, it is the “first” engineer! In particular, cells can be modelled as an adaptive thermal and chemical engines which convert energy in one form to another by coupling metabolic and chemical reactions with transport processes [1–5], by consuming irreversibly [6–8] free energy for thermal and chemical processes, transport of matter, energy and ions.

Energy is a thermodynamic property of any system in relation to a reference state, which changes during any process, while its total amount remains constant in relation to the universe, considering it as the system together with its environment. In cells, many processes such as replication, transcription and translation need to convert molecular binding energy, chemical bond hydrolysis and electromagnetic gradients into mechanical work, related to conformational changes and displacements [9]. The biomechanical analysis of DNA has pointed out the connections among forces, thermodynamics, nano-mechanical and electromagnetic behaviour of biological structures and kinetics [10].

Engineering thermodynamics is the science which studies both energy and its best use in relation to the available energy resources with particular regards to energy conversion, including power production, refrigeration and relationships among the properties of matter, including also living matter. So, engineering thermodynamics can be introduced in the mechanobiological and system biological approach in order to improve these sciences by analysing the biosystems also from a thermal point of view: a new engineering science could be considered, the bioengineering thermodynamics. Indeed, the first law of thermodynamics expresses the conservation of energy, while the second law states that entropy continuously increases for the system and its environment and introduces the statistical and informational meaning of global quantities [11–14].

In this paper we develop the bioengineering thermodynamic of biological cells, with particular regards to possible control of the cells growth by a control of the ions transport across the cell membrane. To do so, we consider that cells spontaneously exchange heat, and this heat is related to their biochemical and biophysical behaviour. This wasted heat represents the interaction between the cell and its environment, a sort of “spontaneous communication” towards environment. This interaction is fundamental to developing a thermodynamic study of the cell. Indeed, cells are too complex to understand the contribution of each process to the global result, and the study of cells as black boxes allows us to simplify the analysis by considering only the inflow and outflow balances [15]. Moreover, it is easier to have access to the cell environment than to the living cell itself. These considerations allow us to introduce the bases of the bioengineering thermodynamic approach introduced in the study of the cells:

1. An open irreversible real linear or non-linear system is considered;
2. Each process has a finite lifetime *τ*;
3. What happens in each instant in the range [0,*τ*] cannot be known, but what has happened after time *τ* (the result of the process) is well-known (at least it is sufficient to wait and observe): local equilibrium is not necessarily required;
4. The balance equations are balance of fluxes of energy, mass and ions.

The fundamental quantity used in this analysis is the global entropy [16, 17], related to systems changes, highlighted as the only effective criterion for spontaneity of change in any system, with particular regards to the entropy variation due to irreversibility, named entropy generation [18], which is the result of the global effect of the entropy variation

1. due to the interaction with the environment
2. within the system itself.

The introduction of entropy generation comes from the need to avoid inequalities: entropy results as a state function, so nothing is really produced or generated. Therefore, entropy is nothing more than a parameter characterising the thermodynamic state, and the term due to irreversibility, *S*_*g*_, measures how far the system is from the state that will be attained in a reversible way [12]. It is always *S*_*g*_ ≥ 0.

Recently, it has been highlighted that any effect in Nature is always the consequence of the dynamic balances of the interactions between the real systems and their environments [12] and the real systems evolution is always related to the decrease of their free energy, in the least time [19–21]. So, bioengineering thermodynamics is based just on two fundamental concepts of physics: interactions and flows. The result is the analytical formulation of flow-based analysis in thermodynamics, which can play the role of a “rallying point” of the different modelling approach to biosystems. Indeed, if we consider natural systems we can highlight that they are always open systems, which means that they can exchange heat and mass with their environment. So, the interaction with the environment is a fundamental concept for the thermodynamic analysis.

We consider the environment as a thermostat and the system, together with its environment, is an adiabatic closed system [18]. But, for an adiabatic close system, the total entropy, defined as:

1 $$ dS={d}_iS+{d}_eS $$

it always increases, as a consequence of the second law [18]. In relation (1) *dS* is the variation of the total entropy elementary, *d*_*e*_*S* is the entropy variation for interaction between the open system considered and its environment, and *d*_*i*_*S* is the entropy variation due to irreversibility, such that:

2 $$ \frac{dS}{dt}\ge 0 $$

Now, we can write the relation (1) as [22]:

3 $$ \frac{dS}{dt}={\displaystyle \underset{V}{\int}\left[-\nabla \cdot \left(\frac{\mathbf{Q}}{T}\right)+{\dot{s}}_g\right]dV} $$

where **Q** is the heat flow, *T* is the temperature, *V* is the volume, *t* is the time and *ṡ*_*g*_ is the density of the entropy generation rate. Now, we consider that the stationary states of the open system correspond to the equilibrium states of the adiabatic closed system. Considering the system together with its environment, we are analyzing an adiabatic closed system, so the entropy variation for the volume considered is maximum at the equilibrium [23]:

4 $$ dS=0\kern1em \Rightarrow \kern1em \left[-\nabla \cdot \left(\frac{\mathbf{Q}}{T}\right)+{\dot{s}}_g\right]\kern0.5em =0 $$

and

5 $$ \nabla \cdot \left(\frac{\mathbf{Q}}{T}\right)={\dot{s}}_g $$

This last relation allows us to state that the flows between the open system and its environment cause the entropy generation rate density, so the interaction between system and environment is responsible of irreversibility. But, we cannot state if the cause of changes is the change of the entropy inside the cell or the fluxes across the cell membrane. We can only highlight the relation between changes and fluxes, but this approach doesn’t allow us to establish if are the fluxes to cause entropy changes or if entropy changes causes fluxes.

Now, considering that the entropy generation rate density can be written as [22]:

6 $$ {\dot{s}}_g={\displaystyle \sum_k{\mathbf{J}}_k\cdot {\mathbf{X}}_k} $$

where **J**_*k*_ is the flow of the *k*-th quantity involved in the process considered and **X**_*k*_ is the related thermodynamic force. Now, considering that:

7 $$ \nabla \cdot \left(\frac{\mathbf{Q}}{T}\right)=\mathbf{Q}\cdot \nabla \left(\frac{1}{T}\right)+\frac{1}{T}\nabla \cdot \mathbf{Q}={\displaystyle \sum_k{\mathbf{J}}_k\cdot {\mathbf{X}}_k} $$

the relation (5) becomes:

8 $$ \frac{1}{T}\nabla \cdot \mathbf{Q}={\displaystyle \sum_k{\mathbf{J}}_k\cdot {\mathbf{X}}_k}-\mathbf{Q}\cdot \nabla \left(\frac{1}{T}\right) $$

in agreement with Le Chatelier’s principle [24], for which any change in concentration, temperature, volume, or pressure generates a readjustment of the system in opposition to the effects of the applied changes in order to establish a new equilibrium, or stationary state. It follows that the fundamental imperative of Nature is to consume free energy in least time. Any readjustment of the state of the system can be obtained only by generating fluxes of free energy which entail any process where the system evolves from one state to another.

## Results and discussion

The existence of bioelectric signalling among most cell types suggests a wide field of applicability of these electro-magnetical signals. Here, we provide bioengineering thermodynamic theory that suggest how to explain the effects of energy, mass and ionic flows across cell membranes and, consequently, to control the cell behaviour by a control of ion fluxes.

Living cells are separated from their environment by the lipid bilayer membrane, which presents a different concentration of specific ion species on both sides. As a consequence, a charge separation across the membrane is generated by the electro-diffusion of ions down their electrochemical gradient. These ions move into a negative (inside the cell) membrane potential of around −70 to −100 mV. The hydrophobic component of the lipid bilayers behaves as a capacitor dielectric, which maintains the ionic gradients across the membrane; in some instances, the action of ATP-driven ionic pumps supports this effect by separating the charges. The cell function is regulated by the membrane proteins, sensitive to electric field; indeed, changes in the electric field are transduced into a conformational change that accomplishes the function of the membrane protein with consequences for the regulation of cell functions. The charged species, their arrangements, the local field strength, charges and dipoles disposition and movements can vary with the result of changing the electric field which is tranduced into a conformational change related to the protein functions themselves [32].

These considerations suggest that control and regulation of the membrane’s electric field could represent a new approach to therapies against diseases such as cancer. To understand how to control the fluxes across the membrane we consider the concentration of the ions on the opposite sides of the membrane [33]:

9 $$ {c}_{outside}={c}_{inside}\kern0.5em  \exp \left(\frac{\varPhi_{outside}-{\varPhi}_{inside}}{RT}\right) $$

where *c* is the molar concentration of the chemical species, *R* is the universal constant of gas, *T* is the temperature and Φ is the electric potential energy. As a consequence of this concentration difference the cell can move the ions, and change the pH inside and outside its membrane. The ion drift velocity *v*_*drift*_ across the cell membrane can be obtained by using the classical kinetic theory [34] as:

10 $$ {v}_{drift}=\frac{Ze}{m}\kern0.5em \frac{\varphi }{d}\kern0.5em {\tau}_{drift} $$

where *Ze* is the electric charge of the ion, *m* is the ion mass, *ϕ* is the electric potential across the membrane, *d* is the length of the membrane and *τ*_*drift*_ is the mean time between two collisions [33]:

11 $$ {\tau}_{drift}=\frac{m\sigma }{n{(Ze)}^2} $$

where *σ* is the electric conductivity. Consequently, an electric current *I* occurs for each ion *i* = H^+^, Na^+^, K^+^, Ca^2+^, Cl^−^, Mg^2+^, etc.:

12 $$ {I}_i={n}_iA{Z}_ie{v}_{drift} $$

where *A* is the mean surface area of the membrane. Now, considering the equivalent RC electric circuit for a membrane it is possible to state that the resonant frequency for such a circuit results in (2π*RC*)^−1^, where *R* is the electric resistivity for the ion considered and *C* is the membrane capacity.

It follows, that if we want to control the cross-membrane flux we must impact the current. The easier physical way to interact with a current is to use an electromagnetic wave of the resonant frequency for the membrane, in relation to the ion considered, with its amplitude being related to the entropy generation as just obtained in Ref. [25–30].

**Fig. 1:**
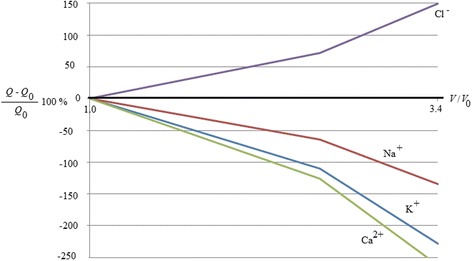
Effect of ions flux control on the energy required by cells to growth in natural conditions

**Fig. 2:**
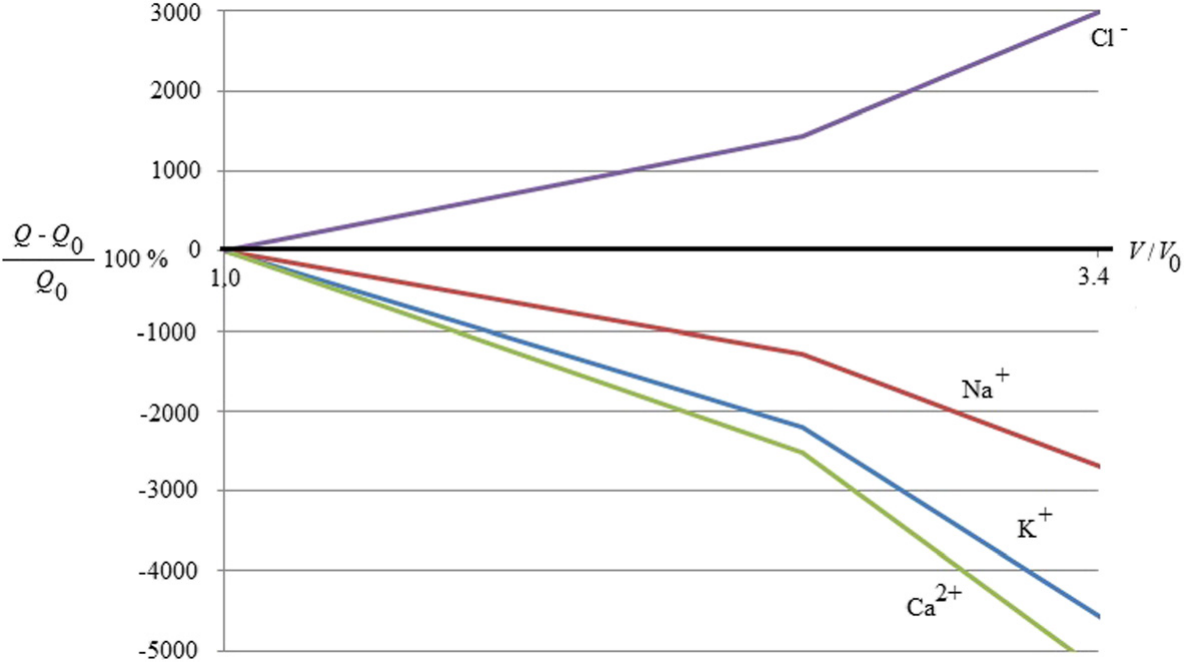
Effect of ions flux control on the energy required by cells to grow in conditions controlled by electromagnetic field

In Figs. 1 and 2, it is represented an example of this kind of control. Figure 1 represents the natural behaviour of cell requirement of energy to grow. Figure 2 represents the cell requirement of energy by cell to grow when they are inside an electromagnetic field. It represents the ratio between the variation in percentage of the energy used by a cancer in a magnetic field (50 μT, 40 Hz) respect the energy used by a cancer outside of the field, related to the energy used by the cancer outside the field vs the growth of the cancer in terms of volume growth (ratio between the cancer volume during the cancer growth and the initial volume). It has been obtained by using the entropy generation approach described in the following section on methods. It is possible to highlight how the different ions have different effect. The positive ions determines a decreasing of the energy used while the negative ion increase it. So the positive ions determine an opposition to the growth. The more effective ion is Ca^2+^. It means that a control of calcium ion can determine a control of the volume growth of a cancer. Here, the control is suggested by the use of an electromagnetic field. The field induces in the cell a greater use of energy to obtain the same growth.

## Conclusions

Life is an organisational and thermodynamic process that tends towards the maximum conversion of available energy. The biochemical reactions produce or consume external metabolites, and they connect internal metabolites, in constant concentrations in the cells at their steady states. To do so, the cell must exchange energy and matter through its membrane. The fundamental phenomena used by cells to reach their optimality consist of a redistributing of the flow patterns through their metabolic network.

By using the bioengineering thermodynamics, it has been highlighted how the different ions have different effect on the use of energy by the cell to grow. To do so, a control of the cells behaviours is introduced. Here, an electromagnetic field is used as a control system, but other field could be used. Cells inside and outside an electromagnetic field have been considered. The positive ions determines a decreasing of the energy used by the cancer, such that the cancer cannot grow as outside the field. On the other hand, the negative ion increase the use of energy. It means that a control of ions can determine a control of the volume growth of a cancer. This result can be extended to all the molecular fluxed across the cell membrane, obtaining a possible bioengineering thermodynamic approach to control the cancer growth.

## Methods

The approach previously used is based on the following considerations:

1. The energy lost by a system is gained by the environment, consequently, the information lost by the system is gained by the environment: here the problem is to codify this information;
2. The environment is completely accessible by any observer, so it is easy to collect data on the lost energy of any system;
3. The flows cause entropy generation variations, consequently we can evaluate the entropy generation to obtain information to the flows, even when we are unable to evaluate the flows themselves;
4. The entropy generation is a global quantity, so we can obtain global information on the cells, but from a biomedical point of view just the global cells behaviour is the useful information.

Biological systems are very interesting because they are able to adapt to the variation of environmental conditions; indeed, cells attain their “optimal” performance by a selection process driven by their environmental interactions. The resultant effect is a redistribution of energy, ions and mass flows in their metabolic network, by using regulatory proteins.

The bioengineering thermodynamic approach to biological systems consists in the analysis of the biological optimization process realized by Nature. It is no more than the classical and engineering thermodynamic analysis of the steady-state flux distribution, which, for a cell, are no more than the metabolic flows. So, starting from Equation (1) and considering the second law for the open systems [18]:

13 $$ {S}_g={\displaystyle \underset{0}{\overset{\tau }{\int }}{\dot{S}}_gdt=\varDelta S-{\displaystyle {\sum}_i\frac{Q_1}{T_i}-}}{\displaystyle \underset{0}{\overset{\tau }{\int }}\left({\displaystyle \sum_{in}{G}_{in}{s}_{in}-}{\displaystyle \sum_{out}{G}_{out}{s}_{out}}\right)dt} $$

where *Q* is the heat exchanged, *T* is the temperature of the thermal source, *s* is the specific entropy, *G* is the mass flow and *τ* is the lifetime of the process. But, for any open system, the entropy balance in a local form results [22]:

14 $$ \frac{\partial s}{\partial t}+v\nabla \cdot \left[\frac{\mathbf{Q}}{T}+{\displaystyle {\sum}_i{\rho}_i{s}_i\left({\dot{\mathbf{x}}}_i-{\dot{\mathbf{x}}}_B\right)}\right]=v\sigma $$

where *s* = *S*/*m*, is the specific entropy, *S* is the entropy, *σ* is the entropy production density, *v* is the specific volume, **Q** is the heat flow, ẋ_*i*_ is the relative velocity in relation to the centre of mass reference, and ẋ_*B*_ is the centre of mass velocity. Now, considering that [22]:

15 $$ \begin{array}{l}T\frac{ds}{dt}=\frac{du}{dt}+p\frac{dv}{dt}-{\displaystyle \sum_i{\mu}_i\frac{d{c}_i}{dt}}\\ {}\frac{du}{dt}=\frac{dq}{dt}-p\frac{dv}{dt}-v\boldsymbol{\Pi} :\nabla {\dot{\mathbf{x}}}_B+v{\displaystyle \sum_k{\mathbf{J}}_k\cdot {\mathbf{F}}_k}\\ {}\frac{dq}{dt}=-v\nabla \cdot {\mathbf{J}}_q\\ {}\frac{d{c}_i}{dt}=-\frac{1}{v}\nabla \cdot {\mathbf{J}}_i+\frac{1}{v}{\displaystyle \sum_j{\nu}_{ij}{J}_j}\end{array} $$

where *s* is the specific entropy, *u* is the internal specific energy, *v* is the specific volume, *p* is the pressure, *μ*_*i*_ are the chemical potentials, *c*_*i*_ is the concentrations, *T* is the temperature, *d*/*dt* = ∂/∂*t* + ẋ_*B*_ ⋅ ∇, *q* is the heat per unit mass, **Π** = **P** – *p***I** with **Π** total pressure tensor, *p* hydrostatic pressure and **I** identity matrix of which the elements are *I*_*jk*_ = *δ*_*jk*_ = 1 if *j* = *k* and 0 in the other cases, **a**:**b** = Σ_*ij*_*a*_*ij*_*b*_*ji*_ is the product between two tensors **a** and **b**, **J**_*k*_ = *ρ*_*k*_ (**ẋ**_i_ − ẋ_*B*_) is the diffusion flows and **F**_*k*_ are the forces, *J*_*j*_ is the chemical reaction rate of the *j*-th chemical reaction and *ν*_*ij*_ are quantities such that if they are divided by the molecular mass of the *i*-th component they are proportional to the stoichiometric coefficients. Now, introducing the electro-chemical affinity Ã = A + *Z* Δ*ϕ* related also to pH variation and the electric field variation, with A_*j*_ = Σ_*k*_*ν*_*kj*_*μ*_*j*_, *Z* the electric charge per unit mass, *ϕ* the electrostatic potential, the relation (AA) holds [25–28]:

16 $$ \begin{array}{c}{S}_g={\displaystyle \underset{V}{\int }dV\left(-{\displaystyle \underset{o}{\overset{\tau_1}{\int }}\frac{v}{T^2}{\mathbf{J}}_q\cdot \nabla Tdt}-{\displaystyle \underset{o}{\overset{\tau_2}{\int }}v{\displaystyle \sum_k{\mathbf{J}}_k\cdot \nabla \left(\frac{\mu_k}{T}\right)dt}}-{\displaystyle \underset{0}{\overset{\tau_3}{\int }}\frac{v}{T}\boldsymbol{\Pi} :\nabla {\dot{\mathbf{x}}}_Bdt}-{\displaystyle \underset{0}{\overset{\tau_4}{\int }}\frac{v}{T}{\displaystyle \sum_j{J}_j{\mathrm{A}}_j}}+{\displaystyle \underset{0}{\overset{\tau_5}{\int }}\frac{v}{T}{\displaystyle \sum_k{\mathbf{J}}_k\cdot {\mathbf{F}}_k}}\right)}=\\ {}={S}_{g,tf}+{S}_{g, dc}+{S}_{g,vg}+{S}_{g,cr}+{S}_{g,de}\end{array} $$

where [25–27]:

1. *S*_*g,tf*_ is the entropy generation due to the thermal flux driven by temperature difference;
2. *S*_*g,dc*_ is the entropy generation due to the diffusion current driven by chemical potential gradients, with $\tilde{\mu}$ = *μ* + *Z *ϕ electrochemical potential, *μ* chemical potential;
3. *S*_*g,vg*_ is the entropy generation due to the velocity gradient coupled with viscous stress;
4. *S*_*g,cr*_ is the entropy generation due to the chemical reaction rate driven by affinity, always positive;
5. *S*_*g,de*_ is the entropy generation due to the dissipation due to work by interaction with the environment;

and *τ*_*i*_, *i* ∈ [1, 5], are the lifetimes of any process and the volume of the cell is evaluated by a characteristic length, in transport phenomena usually considered the diameter of the cell approximated as the diameter of a sphere *L* = (6*V*/*π*)^1/3^ = 2*r*, with *r* being the cell radius;

1. the mean environmental temperature can be assumed as *T*_0_ = 310 K and the mean cell temperature has been estimated to be *T*_0_ + Δ*T*. The quantity Δ*T* would be experimentally evaluated for different cells lines in relation to their metabolism;
2. the internal energy density results in *u* = 3.95 × 10^7^ Jm^−3^, being calculated as the ratio between the ATP energy, *U* = 3 × 10^−7^ J and the mean value of the cell inside the human body, *V* = 7600 μm^3^. It must be emphasized that this is an approximation because the cell volume inside the human body is in the range of 200–15000 μm^3^;
3. the thermal molecular mean velocity inside the cytoplasm is considered to be = 5 × 10^−5^ m s^−1^;
4. the membrane volume is calculated with ${\mathrm{V}}_{\mathrm{m}}=\frac{4}{3}{\uppi \mathrm{r}}^3\hbox{-} \frac{4}{3}\uppi {\left({\mathrm{r}\hbox{-} \mathrm{d}}_{\mathrm{e}}\right)}^3=\frac{4}{3}{\uppi \mathrm{r}}^3\hbox{-} \frac{4}{3}\pi {\left(r\hbox{-} 0.2r\right)}^3=0.992V$ being *d*_*e*_ = 0.2 *r*;
5. the chemical potential gradient can be approximated through the ratio between the mean value of the chemical potential *μ* = 1.20 × 10^−9^ J kg^−1^ and the membrane length *d*_*m*_ = 0.01 μm, with the mean density being *ρ* = 1000 kg m^−3^;
6. the viscosity is taken to be 6.91 × 10^−3^ N s m^−2^;
7. *η* ~ 2.07 × 10^−3^ N s m^−2^ at 30 °C;
8. ẋ_*B*_ is set as 3.0 × 10^−6^ m s^−1^;
9. *τ*_1_ is the time related to the *thermal* flow driven by temperature difference. It can be assessed considering that the time constant of the thermal transient for heat conduction is *τ*_*cv*_ ≈ *pcV*/(*hA*) with *ρ*  ≈ 1000 kg m^−3^ density, *V* the cell volume, *A* the external cell surface, *c* ≈ 4186 J kg^−1^K^−1^ specific heath, and *h* the convection heat transfer coefficient evaluated as: *h* ≈ 0.023*Re*^0.8^*Pr*^0.35^*λ*/*L*,where *λ* ≈ 0.6 W m^−1^K^−1^ of heat conductibility, *L* the characteristic dimension of the cell (here we have considered the diameter), Re ≈ 0.2 the Reynolds number and Pr ≈ 7 the Prandtl number. The process would have occurred in a time *τ*_1_ ≈ 5 *τ*_*cv*_. For human cells this value can be considered in the range 15–269 ms;
10. *τ*_2_ is the time related to the diffusion current driven by chemical potential gradients. It can be evaluated as *τ*_2_ ≈ *d*/*D*, with *d* = 0.01 μm, i.e., the length of the membrane, and *D* being the diffusion coefficient. Considering that the diffusion coefficient of glucose is approximately 10^−9^ m^2^s^−1^ it follows that *τ*_2_ ≈ 10 s;
11. *τ*_3_ is the time related to the velocity gradient coupled with viscous stress. This time can be evaluated as the propagating time of a mechanical wave on the surface of the cell ${\tau}_3\approx \frac{2\pi r}{c}$ with *c* ~ 1540 m s^−1^ the sound velocity, considered to be the same in biological tissue;
12. *τ*_4_ is the time related to the chemical reaction rate driven by affinity and it can be evaluated considering the magnitude order of a chemical reaction in a cell (~10^−7^ mol s^−1^l^−1^). Moreover, we consider that the moles number is proportional to the density of the chemical species (for glucose 1540 kg m^−3^) and the volume of the cell itself. It follows that this time is in the range 17–1283 ns;
13. *τ*_5_ is the time related to the dissipation due to work by interaction with the external forces. It depends on the interaction considered;
14. *L* is a characteristic length, introduced as usually done in transport phenomena.

An experiment has been developed to obtain also a direct proof [31]. Therefore the spontaneous heat exchanged by the cell represents the interaction or the spontaneous communication between the cell and its environment. The proposed thermodynamic theory predicts that the temperature difference between cells with distinct metabolic characteristics can be amplified by an altered interaction with the external environment, due to the entropy generation term related to the interaction of the system with the external fields. The experiments carried out on cells exposed to low frequency electromagnetic waves consolidate the thermodynamic approach. Indeed, through infrared thermography an adimensional number, maned thermal dispersion index, was evaluated. This adimensional number represents the inability of the cells to fit their thermal power to environmental changes. Primary fibroblasts display a high dispersion index, with a maximal value of 800 % vs NIH3T3 immortalized line, which means that the primary fibroblasts adjust more efficiently their thermal production or dissipation than the NIH3T3 cells. This significant difference implies that, when exposed to selected environmental conditions, transformed cells dissipate heat more slowly than their normal counterpart. The results of this experimental approach demonstrate that selecting environmental conditions it is possible to appreciate distinct cellular phenotypes; these differences can be evaluated by thermal dispersion patterns measured by infrared thermography. The experiment confirmed the bioengineering thermodynamics theoretical results.

The results obtained can be improved by considering other approach to bioengineering thermodynamics devoted to the study of organization in living systems and by linking each others. Indeed, evolution over the long term requires a constant generation of new alternative forms, a biological behavior named mutation [35]. The cooperative effect of mutation and selection consists in different processes on different time scales:

1. Microevolution: changes within a natural populations, in the composition of populations due to mutation and natural selection. It occurs on a time scale of generations, it represents an adaptive change;
2. Macroevolution: changes between populations, in the composition of lineages due to speciation and extinction. It occurs in a geological time scale.

Macroevolution has its origin in microevolution as the result of natural selection acting on genotypic and phenotypic variation [35–38]. These natural processes can be described by introducing mathematical models, based on two thermodynamic actions [35, 39]:

1. The acquisition of resources from the external environment and its conversion into energy storage;
2. The transformation of the metabolic energy into useful work.

The bases of these processes is the interaction between bio-system and environment [40]. This brings to non equilibrium states, and the mathematical formalisms developed to the biosystems analysis was the dynamical systems, based on the studies of Bowen [41], Ruelle [42] and Sinai [43], who provided new perspectives in the analysis of far from equilibrium systems by the discovery of certain connections between non-equilibrium statistical mechanics and the ergodic theory of dynamical systems. In this context the fundamental concept is the entropy and just this concept represents the link between the dynamical systems approach and the thermodynamic approach here developed. Indeed, following Ruelle [44], considering a classical system with isokinetic time evolution described by the equation:

17 $$ \frac{d}{dt}\left(\begin{array}{c}\hfill \mathbf{p}\hfill \\ {}\hfill \mathbf{q}\hfill \end{array}\right)=\left(\begin{array}{c}\hfill \boldsymbol{\upxi} -\alpha \mathbf{p}\hfill \\ {}\hfill \mathbf{p}/m\hfill \end{array}\right)\iff \frac{d\mathbf{x}}{dt}={\mathbf{F}}_{\boldsymbol{\upxi}}\left(\mathbf{x}\right) $$

with

18 $$ \mathbf{x}=\left(\begin{array}{c}\hfill \mathbf{p}\hfill \\ {}\hfill \mathbf{q}\hfill \end{array}\right)\kern2.5em \mathrm{and}\kern2.5em {\mathbf{F}}_{\boldsymbol{\upxi}}\left(\mathbf{x}\right)=\left(\begin{array}{c}\hfill \boldsymbol{\upxi} -\alpha \mathbf{p}\hfill \\ {}\hfill \mathbf{p}/m\hfill \end{array}\right) $$

**p**∈***R***^*N*^ and **q**∈***R***^*N*^ momentum and position respectively, **ξ** a nongradient time independent force, *m* mass and (−*α***p**) the isokinetic thermostat mathematical expression with *α* defined as:

19 $$ \alpha \left(\mathbf{x}\right)=\frac{\mathbf{p}\cdot \boldsymbol{\upxi} \left(\mathbf{q}\right)}{\mathbf{p}\cdot \mathbf{p}} $$

so that [44]:

20 $$ \frac{d}{dt}\left(\frac{\mathbf{p}\cdot \mathbf{p}}{2m}\right)=0 $$

Under these conditions Ruelle defined the entropy increment as [44]

21 $$ S\left(\boldsymbol{\upxi} +\varDelta \boldsymbol{\upxi} \right)={\displaystyle \underset{0}{\overset{\infty }{\int }}dt{\displaystyle \underset{-\infty }{\overset{t}{\int }}d\tau {\rho}_{\boldsymbol{\upxi}}\left({\nabla}_{\mathbf{x}}\left(d\mathbf{x}\circ {f}_{{\boldsymbol{\upxi}}_0}^{t-\tau}\right)\cdot {\delta}_{\tau}\mathbf{F}\left(\mathbf{x}\right)\right)\varPhi \left(\mathbf{x}\right)}} $$

with *δ*_*τ*_**F** is a time-dependent small perturbation of **F**, ${\rho}_{\boldsymbol{\upxi}}\left({\nabla}_{\mathbf{x}}\left(d\mathbf{x}\circ {f}_{{\boldsymbol{\upxi}}_0}^{t-\tau}\right)\cdot {\delta}_{\tau}\mathbf{F}\left(\mathbf{x}\right)\right)$ probability distribution, *f*_**ξ**_^*t* − *τ*^ the solution of the equation (18) at the time *t*-*τ* corresponding to the initial conditions **ξ**_0_, Φ(**x**) = (*N* - 1)*α*. Then, Denbigh [18, 45] expressed the fundamental processes of living systems, introducing an entropy approach:

22 $$ dS=d{S}_{\mathrm{int}}+d{S}_{\mathrm{ext}} $$

where *dS* is the total entropy elementary variation, *dS*_int_ is the entropy elementary production within the system due to its metabolism of ingested exergy and *dS*_ext_ is the entropy exchange with the environment. Entropy is a path independent state function, and the overall reaction entropy Δ*S*_*R*_ can be evaluated by the macroscopic reaction stoichiometry between external metabolites:

23 $$ \varDelta {S}_R={\displaystyle \sum_{i=1}^n{p}_i\varDelta {S}_i}={\displaystyle \sum_{i=1}^n{p}_i{\displaystyle \sum_{l=1}^k{\nu}_l{s}_{li}}}=c{\displaystyle \sum_{i=1}^n{p}_i \ln {p}_i} $$

where Δ*S*_*i*_ = − *c* ln *p*_*i*_ is the entropy of reaction, *s*_*li*_ = (*h*_*li*_ – *g*_*li*_)/*T*, with *h*_*li*_ molar enthalpy and *g*_*li*_ Gibbs molar energy, are the molar entropies of the *k* reactants and products, *ν*_*l*_ are the stoichiometry coefficients, *p*_*i*_ is the probability of the *i*-th mode and *c* is a constant related to the numbers of elementary modes and on their reaction entropies. It represents the state of the fully evolved metabolic network [46]. When the living systems increase in organization, they increase their entropy and, far from equilibrium, they have a high exergy content [47]; indeed, considering two systems with the same mass and the same chemical composition, the one, that has a large amount of organization, has also higher exergy content. During their evolution, the living systems, and also ecosystems, increase their structure in organization, which is a working information useful for resilience and integrity, and also their efficiency in converting exergy to entropy, in order to reduce the applied exergy gradient, while their internal entropic state continue to decrease [48, 49]. Then, while *dS*_int_ is always positive defined (*dS*_int_ ≥ 0), *dS*_ext_ can have any sign.

The inner entropy generation rate *σ* is defined as the local first time derivative of the [50] internal component of the entropy:

24 $$ \sigma =\frac{d{S}_{\mathrm{int}}}{dt} $$

If the irreversible processes are sufficiently slow, the Gibbs equation can be applied to any subsystem [50]:

25 $$ TdS=dU+pdv-{\displaystyle \sum_i{\mu}_id{n}_i} $$

and the entropy can be expressed in terms of fluxes *J*_*i*_ and conjugated generalized forces *X*_*i*_ [50]:

26 $$ T\sigma ={\displaystyle \sum_i{J}_i{X}_i} $$

The non-equilibrium stationary states, which are the states whose variables are independent of time, play a fundamental role in the irreversible processes. After a characteristic time, the system achieves the equilibrium if no restraints are imposed on it, while if a number of constant restraints are imposed, a steady state is attained [50]. In any steady state the total entropy is independent of time, consequently:

27 $$ \frac{dS}{dt}=\frac{d{S}_{\mathrm{int}}}{dt}+\frac{d{S}_{\mathrm{ext}}}{dt}=0\Rightarrow \frac{d{S}_{\mathrm{ext}}}{dt}=-\frac{d{S}_{\mathrm{int}}}{dt} $$

but

28 $$ \frac{d{S}_{\mathrm{int}}}{dt}\ge 0\Rightarrow \frac{d{S}_{\mathrm{ext}}}{dt}\le 0 $$

and it is possible to argue that the entropy generation rate in a stationary system must be compensated by the liberation of entropy to the surroundings. This means also that non-equilibrium steady states cannot occur in isolated systems because these last systems do not allow exchange of entropy between the systems and the surroundings [8]. Prigogine proved that [51–53]:

29 $$ d\sigma \le 0\Rightarrow \frac{d^2{S}_{\mathrm{int}}}{d{t}^2}\le 0 $$

On the use of the Prigogine’s results there is little doubt that a mature organism may reached a stationary state; indeed, the homeostasis of all self regulating systems is interpreted as tendency to return from a perturbed state to that of highest stability compatible with biological constraints [50].

Moreover, considering an irreversible and open system, it is composed by *N* elementary volumes. Every *i*-th element of this system is located by a position vector **x**_*i*_, it has a velocity ẋ_*i*_, a mass *m*_*i*_ and a momentum **p**_*i*_ = *m*_*i*_ẋ_*i*_. The total mass of the system is *m* = ∑_*i*_*m*_*i*_ and its density is *ρ = m*/*V* with *V* = ∑_*i*_*V*_*i*_ total volume. The position of the centre of mass is **x**_*B*_ and its velocity results ẋ_*B*_ = ∑_*i*_*m*_*i*_ẋ_*i*_/*m*, while the diffusion velocity is **u**_*i*_ = ẋ_*i*_ − ẋ_*B*_. The total mass of the system is conserved, so the following relation $\dot{\rho}+\rho \nabla \cdot {\dot{\mathbf{x}}}_B=0$ is satisfied together with its local expression ${\dot{\rho}}_i+\rho \nabla \cdot {\dot{\mathbf{x}}}_i=\rho {\varXi}_i$, related to the density of the *i*-th elementary volume of density *ρ*_*i*_ and a source Ξ generated by matter transfer, chemical reactions or thermodynamic transformations. For an open system, as just described in macroscopic way, the equation of the entropy balance is [22]:

30 $$ \begin{array}{l}\frac{\partial s}{\partial t}+v\nabla \cdot {\mathbf{J}}_S=\dot{s}\\ {}\dot{s}=v\sigma \end{array} $$

where *s* = *S*/*m*, is the specific entropy, *S* entropy, *σ* the density of the entropy generation rate, *v* the specific volume and **J**_*S*_ is the entropic flux defined as:

31 $$ {\mathbf{J}}_S=\frac{\mathbf{Q}}{T}+{\displaystyle {\sum}_i{\rho}_i{s}_i\left({\dot{\mathbf{x}}}_i-{\dot{\mathbf{x}}}_B\right)} $$

with **Q** heat flux.

Any dynamical state of this system can be described by the 3*N* canonical coordinates {**x**_*i*_ ∈ *R*^3^, *i* ∈ [1,*N*]} and their conjugate momenta {**p**_*i*_ ∈ *R*^3^, *i* ∈ [1,*N*]}. The 6*N* − dimensional space spanned by{(**p**_*i*_,**x**_*i*_), *i* ∈ [1,*N*]} is the phase space Ω of the open system considered. Any point **q**_*i*_ = (**p**_*i*_,**x**_*i*_), **q**_*i*_ ∈ *R*^6*N*^ in the phase space Ω, represents a state of the entire *N* − elements system [54]. Any family {ξ(*t*), *t* ∈ R} is called stochastic process in the phase space Ω and it can be represented by a family of equivalent classes of random variables ξ(*t*) on Ω, {*γ*(σ(*t*)) : *t* ∈ R}. The point function *γ*(**q**(*t*)) is called trajectory of the stochastic process ξ(*t*): a description of a physical system in terms of a trajectory of a stochastic process corresponds to a point dynamics, while its description in terms of equivalent classes of trajectories and their associated probability measure corresponds to an ensemble dynamics [55]. So it is considered a non-equilibrium system moving in the Ω-space between two states, which are in two elementary cells of a given partition of the phase space. We use the concept of path of classical mechanics: if the motion of the system is regular, or if the phase manifold has positive or zero Riemannian curvature, there will be only a fine bundle of paths which track each other between the initial and the final cells [13]. For a system in chaotic motion, or when the Riemannian curvature of the phase manifold is negative, two points indistinguishable in the initial cell can separate from each other exponentially [54]. Then, between two given phase cells, there may be many possible paths *γ*_*k*_, *k* ∈ [1,*ω*] with *ω* number of all the paths, with different travelling time *t*_*γk*_ of the system and different probability *p*_*γk*_ for the system to take the path *k*, called path probability distribution [56–59]. It is considered an ensemble of a large number *L* of identical systems, all moving in the phase space from two cells with *ω* possible paths, and *L*_*k*_ systems travelling on the path *γ*_*k*_. The probability *p*_*γk*_ that the system take the path *γ*_*k*_ is defined as usual by *p*_*γk*_ = *L*_*k*_/*L*. If *ω*_*k*_ = 1 then *p*_*γk*_ = 1. By definition, *p*_*γk*_ is the transition probability from the two states considered. These trajectories must be the paths minimizing action according to the principle of least action [54]. Since 1962, Jaynes argued that Gibbs’ formalism of equilibrium statistical mechanics could be generalised in a statistical inference theory for non-equilibrium systems [60]. Jaynes developed the non-equilibrium statistical mechanics for the stationary state constraint on the basis of maximum entropy; his approach consists of maximising the path Shannon information entropy written for the path, *S*_*I*_ = − Σ_*γ*_*p*_*γ*_ ln *p*_*γ*_, with respect to *p*_*γ*_ of the path *γ*, with the probability subject to the actual constraints. According to Shannon, ‘the information entropy is the logarithm of the number of the outcomes *i* with non-negligible probability *p*_*i*_’, while in ‘non-equilibrium statistical mechanics it is the logarithm of the number of microscopic phase-space paths *γ* having non-negligible probability *p*_*γ*_’ [60]. Jaynes’ approach consists of finding the ‘most probable macroscopic path realised by the greater number of microscopic paths compatible with the imposed constrained’ [60], in analogy with the Boltzmann microstate counting: ‘paths rather then states are the central objects of interest in non-equilibrium systems, because of the presence of non-zero macroscopic fluxes whose statistical description requires considering the underlying microscopic behaviour over time’ [60] which implies that ‘the macroscopic behaviour is reproducible under given constraints’ and it is ‘characteristic of each of the great number of microscopic paths compatible with those constraints’ [60]. Following this approach and these considerations, the statistical expression of the entropy generation has been written as [56–59]:

32 $$ {S}_g=-{k}_B{\displaystyle \sum_k{p}_{\gamma k} \ln }{p}_{\gamma k} $$

It can be also interpreted as the missing information necessary for predicting which path a system of the ensemble takes during the transition from a state to another.

In the theory of probability the stochastic order is introduced. Two random variables *X* and *Y* are in stochastic order if there exists a random variable *Z* and functions *ψ*_1_ and *ψ*_2_ such that *X* = *ψ*_1_(*Z*) and *Y* = *ψ*_2_(*Z*), with *ψ*_1_(*Z*) ≤ *ψ*_2_(*Z*) [61]. Now, the set of paths {*γ*_*k*_, *k* ∈ [1,*ω*]} is considered, with *ω* number of all the paths between two thermodynamic states, represented by two points in the phase space. It is possible to define a stochastic order among the paths, saying that a path *γ*_*i*_ is stochastically smaller than a path *γ*_*j*_ if its probability *p*_*γi*_ is smaller that the probability of the other path, *p*_*γj*_ [13]:

33 $$ {\gamma}_i{<}_{ST}{\gamma}_j\kern1.5em \mathrm{if}\kern2em {p}_{\gamma i}<{p}_{\gamma j} $$

So, the probability of a path can be expressed in term of the first order differential of the entropy generation respect to the probability itself, as follows [13]:

34 $$ {p}_{\gamma i}= \exp \left(-\frac{1}{k_B}\frac{\partial {S}_g}{\partial {p}_{\gamma i}}-1\right) $$

But, in the analysis of the complex systems, it was highlighted how chaotic and fractal behaviour are very widespread in nature: any numerical evaluation based on accessible states in the phase space is incomplete because of the rejected, singular or inaccessible points [56]. The basis of the incomplete information is that a part of information on complex system may not be completely accessible. The consequence is that irreversibility is the physical model by which thermodynamic phenomena can be completely described [54]. The related information is incomplete because, for complex systems, it occurs that ∑_*j* = 1_^*ν*^*p*_*j*_ = *θ* ≤ 1, with *ν* number of accessible or accountable states, smaller of the total number of states, and *θ* incompleteness of the treatment and linked to the nature of the system, consequence of the partial knowledge of the dynamics or of the inaccessible states of the system itself [54]. Non-statistical mechanics replaces the complete probability normalisation by:

35 $$ {\displaystyle \sum_{j=1}^{\nu }{p}_j^{\vartheta }}=1 $$

with *ϑ* incompleteness parameter such that *ϑ* = 1 if the probability distribution is complete. It can be related to the incompleteness *θ* by the following relation [13]:

36 $$ {\displaystyle \sum_{j=1}^{\nu -1}{p}_j^{\vartheta }}+{\left(\theta -{\displaystyle \sum_{j=1}^{\nu -1}{p}_j}\right)}^{\vartheta }=1 $$

The phase space cells, which represent the stationary states, was proven to form a subset of all the cells on which the evolution acts as a one-cycle permutation: this kind of ergodicity has been defined ergodicity for irreversibility [54]. Moreover, in non-equilibrium transformation, the volume of the phase space was proven to contract indefinitely [54]. Recently, considering the expression for the probability *p*_*γi*_ of a path *γ*_*i*_ and the statistical results on the entropy generation [54], it was proven that [13]:

37 $$ \frac{\partial {S}_g}{\partial {p}_{\gamma i}}\le \frac{\partial {S}_g}{\partial {p}_{\gamma j}}\kern1.5em \mathrm{if}\kern1.5em {p}_{\gamma i}\le {p}_{\gamma j} $$

which means that the paths are statistically ordered. The stochastic order of the path proves that the evolution of the bio-systems is related to their irreversibility and the quantity useful to evaluate the allowed paths and their probability is the entropy generation. Consequently, a link between the bioengineering thermodynamic approach proposed and the dynamical system approach is obtained.

## References

[1] Demirel Y, Sandler SI (2002). Thermodynamics and bioenergetics. Biophys Chem.

[2] Toussaint O, Schneider ED (1998). The thermodynamic and evolution of complexity in biological systems. Comp Biochem Physiol A.

[3] Caplan SR, Essig A (1983). Bioenergetics and Linear Nonequilibrium Thermodynamics, The Steady State.

[4] Lucia U (2014). Entropy generation approach to cell systems. Physica A.

[5] Lucia U (2013). Molecular machine as chemical-thermodynamic devices. Chem Phys Lett.

[6] Salerian AJ, Saleri NG (2008). Cooling core body temperature may slow down neurodegeneration. CNS Spectr.

[7] Katchalsky A, Curran PF (1967). Nonequilibrium thermodynamics in biophysics.

[8] Lucia U (2012). Irreversibility in biophysical and biochemical engineering. Physica A.

[9] Bustamante C, Chemla YR, Forde NR, Izhaky D (2004). Mechanical processes in biochemistry. Annu Rev Biochem.

[10] Hudspeth A, Choe Y, Mehta A, Martin P (2000). Putting ion channels to work: mechanoelectrical transduction, adaptation and amplication by hair cells. Proc Natl Acad Sci.

[11] Lucia U (2009). Irreversibility, entropy and incomplete information. Physica A.

[12] Lucia U (2013). (2013b) Stationary open systems: A brief review on contemporary theories on irreversibility. Physica A.

[13] Lucia U (2013). Thermodynamic paths and stochastic order in open systems. Physica A.

[14] Lucia U (2013). Thermodynamics and cancer stationary states. Physica A.

[15] Lucia U (2014). Transport processes in biological systems: tumoral cells and human brain. Physica A.

[16] Denbigh KG (1989). Note on entropy, disorder and disorganization. Brit J Phil Sci.

[17] Denbigh KG (1989). The many faces of irreversibility. Brit J Phil Sci.

[18] Bejan A (2006). Advance engineering thermodynamics.

[19] Lucia U (2015). Some considerations on molecular machines and Loschmidt paradox. Chem Phys Lett.

[20] Lucia U (2015). A link between nano- and classical thermodynamics: dissipation analysis (the entropy generation approach in nano-thermodynamics). Entropy.

[21] Lucia U (2015). Entropy production and generation: clarity from nanosystems considerations. Chem Phys Lett.

[22] de Groot SG, Mazur P (1984). Non-equilibrium thermodynamics.

[23] Zemansky MW (1966). Heat and thermodynamics.

[24] Atkins PW (1993). The elements of physical chemistry.

[25] Lucia U (2014). Entropy generation and cell growth with comments for a thermodynamic anticancer approach. Physica A.

[26] Lucia U (2014). Thermodynamic approach to nano-properties of cell membrane. Physica A.

[27] Lucia U (2014). Transport processes and irreversible thermodynamics analysis in tumoral systems. Physica A.

[28] Lucia U (2014). The gouy-stodola theorem in bioenergetic analysis of living systems (Irreversibility in bioenergetics of living systems). Energies.

[29] Lucia U, Ponzetto A, Deisboeck TS (2014). A thermo-physical analysis of the proton pump vacuolar-ATPase: the constructal approach. Sci Rep.

[30] Lucia U, Ponzetto A, Deisboeck TS (2015). A thermodynamic approach to the ‘mitosis/apoptosis’ ratio in cancer. Physica A.

[31] Lucia U, Grazzini G, Montrucchio B, Grisolia G, Borchiellini R, Gervino G (2015). Constructal thermodynamics combined with infrared experiments to evaluate temperature differences in cells. Sci Rep.

[32] Tuszynski JA, Kurzynski M (2003). Introduction to molecular biophysics.

[33] Newman J (2008). Physics of the life sciences.

[34] Kittel C, Kroemer H (1980). Thermal physics.

[35] Demetrius LA, Gundlach VM (2014). Directionality theory and the entropic principle in natural selection. Entropy.

[36] Mayr E (2002). What evolution is.

[37] Mayr E (1976). Evolution and the diversity of life.

[38] Demetrius L (1997). Directionality principles in thermodynamics and evolution. Proc Natl Acad Sci U S A.

[39] Lehninger A (1965). Bioenergetics.

[40] Lewontin RC, Bendall DS (1983). Gene, organism and environment. Evolution from molecules to men.

[41] Bowen R (1975). Equilibrium states and the ergodic theory of anosov diffeomorphisms. Vol. 470 lecture notes in math.

[42] Ruelle D (1978). Thermodynamic formalism. Vol. 5 encyclopedia of mathematics and its applications.

[43] Sinai YG (1972). Gibbs measures in ergodic theory. Russ Math Surv.

[44] Ruelle D (2003). Extending the definition of entropy to nonequilibrium steady states. Proc Natl Acad Sci U S A.

[45] Denbigh KG (1989). Note on entropy, disorder and disorganization. Brit J Phil Sci.

[46] Sandler SI, Orbey H (1991). On the thermodynamics of microbial-growth processes. Biotech Bioeng.

[47] Swenson R (1989). Emergent attractors and the law of maximum entropy prpduction: foundations to a theory of general evolution. Systems Research.

[48] Brooks DR, Collier J, Maurer BA, Smith JDH, Wiley EO (1989). Entropy and information in evolving biological systems. Biology & Philosophy.

[49] Günther F, Folke C (1993). Characteristics of nested living systems. J Biological Systems.

[50] Katchalsky A, Kedem O (1962). Thermodynamics of flow processes in biological systems. Biophys J.

[51] Glansdorff P, Prigogine I (1971). Thermodynamic theory of structure, stability and fluctuations.

[52] Prigogine I (1947). Etude Thermodynamique des Phénomènes Irrèversibles.

[53] Prigogine I (1961). Introduction to thermodynamics of irreversible processes.

[54] Lucia U (2008). Probability, ergodicity, irreversibility and dynamical systems. Proc Royal Soc A.

[55] Primas H (1999). Basic elements and problems of probability theory. J Sci Explor.

[56] Lucia U (2008). Statistical approach of the irreversible entropy variation. Physica A.

[57] Lucia U (2009). Irreversibility, entropy and incomplete information. Physica A.

[58] Lucia U (2010). Maximum entropy generation and *κ* − exponential model. Physica A.

[59] Lucia U (2007). Irreversibility entropy variation and the problem of the trend to equilibrium. Physica A.

[60] Dewar R (2003). Information theory explanation of the fluctuation theorem, maximum entropy production and self-organized criticality in non-equilibrium stationary states. J Phys A: Math Gen.

[61] Shaked M (2006). Stochastic orders.

